# Stories of self, us, and now: narrative and power for health equity in grassroots community organizing

**DOI:** 10.3389/fpubh.2023.1144123

**Published:** 2023-06-05

**Authors:** Krista A. Haapanen, Brian D. Christens, Hannah E. Freeman, Paul W. Speer, Gavin A. Crowell-Williamson

**Affiliations:** ^1^Department of Human and Organizational Development, Vanderbilt University, Nashville, TN, United States; ^2^Bridge Maryland, Baltimore, MD, United States

**Keywords:** health equity, grassroots community organizing, public narrative, narrative change, narrative construction, narrative intervention, power, empowerment

## Abstract

**Introduction:**

Community organizing initiatives, which build power through cycles of listening, participatory research, collective action, and reflection, have demonstrated the capacity to intervene on, complicate, and resist dominant societal narratives while promoting alternative public narratives focused on shared values and hope for a better future.

**Methods:**

To explore processes of public narrative change and their relationship to community and organizational empowerment, we interviewed 35 key leaders in community organizing initiatives in Detroit, MI and Cincinnati, OH about how narrative change takes place within community organizing practices.

**Results:**

Leaders’ perspectives revealed crucial roles for narrative and storytelling in guiding individual and collective behavior, supporting the development of relationships of trust and accountability, and linking personal and collective experiences to pressing social issues.

**Discussion:**

Findings from this study indicate that systemic change is a labor-intensive process and one that requires the development of leaders (stories of self) and the cultivation of collective structures (stories of us) capable of enacting power to effect change with urgency (stories of now). We conclude by discussing implications of these findings for public narrative interventions and related health equity promotion efforts.

## Introduction

The last two decades have seen a rapid proliferation of scholarship related to health equity and social justice. The now well-established relationship between health and social factors (i.e., the social determinants of health, or SDOH) such as education, housing, and the built environment has paved the way for recognition of more fundamental determinants of health such as governance, policy, and societal norms, values, and beliefs (1). Referred to variously as “root causes” (2) or “causes of causes” (3), these fundamental determinants of health inequity are deeply entrenched asymmetries in social power that create and perpetuate differences in health outcomes (4).

Recently, the notion of *public narrative* has emerged as a focal point in discourses about these fundamental causes of health inequity. Narratives manifest in all expressions of culture, from myths, stories, and social rules to advertisements, works of fiction, museum displays, and language. A *dominant* public narrative is defined by the National Network of Public Health Institutes (NNPHI) as

A belief system, pattern of thought and framework for how we collectively understand each other and the world around us. It helps us determine how we live and respond to the things we experience. It’s a frame that helps us assess experiences and frame beliefs as to what is worthy or right (2021, p. 12).

Dominant narratives inform what we view as natural and normal, or immoral or unjust, thus guiding the behavior of individuals and society (5). A dominant narrative driving health inequity in the U.S., for instance, is that racism only takes the form of overt discrimination or interpersonal bias. This narrative renders invisible all of the covert ways that systems and policies privilege the interests of White people, thus upholding the deeply entrenched and discriminatory practices and policies that reinforce exclusion (6).

The effects of public narrative on identity, perceptions, and behavior make it a promising lever for health equity-focused intervention. Studies of addiction suggest, for instance, that adolescents are influenced by media portrayals of health behaviors such as smoking, indicating that feelings of identification and liking with movie characters may be a more powerful driver for behavior change than traditional data-and evidence-focused health communication (7). This aligns with Bruner’s (8) observation that, in addition to scientific ways of knowing, there are *narrative* ways of knowing that relate to personal experience and the experience of others (9). Narrative intervention for health promotion has largely focused on leveraging this aspect of narrative, “devis [ing] inspiring, coherent, dramatic stories that reach people’s hearts and minds” [(10), p. 8]. Such efforts typically employ expert-driven processes that engage communities mainly in the deployment of the new messages and frames (11).

Although this approach to narrative change can be influential, one weakness is that many of such programmatic interventions tend to focus more on individual behaviors than they do on changing policies, systems, and environments. New narratives are developed by experts with the goal of persuading individuals to change their behavior (9), and use messaging campaigns to disseminate their messages. They therefore target individuals, implicitly suggesting that individuals—not groups or collectives—are what ultimately determine health. Even when policy is the target of these campaigns, narrative change efforts are often focused on getting people on board with a candidate or agenda. Thus, although the desired outcome is structural in nature, the approach is nevertheless individualized.

These weaknesses of programmatic narrative interventions call for different approaches to narrative change. Grassroots community organizing initiatives have a demonstrated ability to change public narratives using community-driven, rather than expert-or researcher-driven, approaches. Community organizing is a process that connects people to collective efforts by bringing local residents together to understand common concerns in their communities, investigate possible solutions to these concerns, and take collective action to change the conditions that create and perpetuate these concerns (12). Through these processes, many residents who are engaged on a voluntary basis develop as community leaders (13). This type of work often takes place through federations of faith-based institutions, for several reasons: they are often some of the most vibrant institutions in low-income communities; regardless of faith traditions and perspectives, they are anchored in values that can support efforts to build collaborations across diverse interests of community members; and they are often deeply invested in the communities in which they are located (14, 15). Some previous studies demonstrate the promise of grassroots community organizing for intervening on and complicating dominant narratives, illustrating how community organizing creates social settings in which dominant cultural narratives can be discussed and challenged, and in which individuals can weave new individual and collective identities that reflect their own lived experience (16, 17).

### Community organizing and public narrative

Unlike narrative change approaches that rely on experts to develop and disseminate new narratives for uptake by the public, grassroots community organizing initiatives weave together the development of new narratives with broader efforts to develop community power. Community power refers to the ability of communities most impacted by structural inequities to develop, sustain, and grow an organized base of people who act together through democratic structures to change systems (18). Contemporary theories of community power and empowerment center the idea that producing sustained changes to inequitable systems requires organized groups of people who can coordinate participation in pursuit of a common goal or purpose (19, 20). Organization, in this view, is among the most important sources of sociopolitical power in modern society (21).

Community organizing initiatives contend that some of the most harmful dominant narratives frame groups of people as politically powerless or apolitical (17). This narrative, when internalized, can create feelings of hopelessness and apathy that form barriers to political action. Gupta (17) illustrates how participants in faith-based community organizing challenge these narratives by engaging in dialogue and collective actions that cultivate their identities as political agents. The organization, she suggests, provides a space in which dominant narratives can be discussed and critiqued, and serves as “a set of tools or resources upon which subjects draw as they construct their political selves and sense of their selves in the world” [(17), p. 18]. Overcoming the barriers to political action, Gupta suggests, involves not just a well-crafted and inspiring story, but organizational settings in which participants can recognize and challenge dominant narratives and construct more agentic political identities. The narratives constructed through these processes, moreover, can sometimes connect local organizing efforts and larger social movements (22).

Through participating in community organizing, people often develop the understanding that prevailing narratives most often reflect the interests and experiences of the powerful, and that these powerful actors have a vested interest in preventing people from identifying shared challenges and acting collectively to address them (13). Narratives that frame individuals as alone in their problems or to blame for their circumstances prevent communities from forming bonds of solidarity, thus hindering the development of community power. Core organizing practices of listening and reflection counteract these narratives by weaving together participants’ personal and collective identities, enabling them to identify shared self-interests and to recognize issues that the organizing initiative can then begin to research for potential future action (23). The sharing of personal stories, combined with critical analysis of power structures, forms the basis for relationships of mutual respect and civic accountability that bind these organizations together (24).

Narrative change also occurs through the organizing practice of critical social analysis, which engages participants in a form of civic learning about the issues and structures that affect them (24). Once social issues of interest to the community are identified, organizing initiatives engage in a critical analysis of the issue focused on identifying structural aspects of these problems and connecting them to personal experiences of those problems. Recognizing the structural origins of personal challenges reframes their solutions, suggesting that collective actions to change structures—rather than individual changes in behavior—are needed to achieve desired changes. Reframing social issues as immoral or unjust, moreover, can yield affective changes conducive to public action, which can in turn reframe issues in broader public debates [e.g. (25),]. Feelings such as urgency, solidarity, and anger can overcome barriers to action such as fear, apathy, and self-doubt. It’s worth nothing that this affective power of narrative is also what has driven approaches often taken in programmatic narrative change initiatives. However, these approaches are rarely developed through grassroots processes that allow participants to link their personal stories with new public narratives.

Ganz (26), a scholar and organizer, developed a framework to describe how public narrative work can take place in grassroots community organizing. According to Ganz, the process of translating values into action consists of integrating a story of *self*, a story of *us*, and a story of *now*. The story of *self* describes why an individual is involved with an issue (“Which experiences led you to get involved?”). The story of *us* involves the process of coming together with others to form a collectivity in pursuit of change on the issue. The story of *now* narrates the urgent change that is needed at this moment. Narrative change work is a constant practice in community organizing; public narratives are constantly reshaped as people change, as new people come in and out of communities and organizations (shifting the “us” element), and as issues are addressed and new ones arise. Leaders are constantly sharing their own public narratives, getting feedback from others in the organization, and refining them. It is through these discursive processes that participants build their sense of self and way of understanding their relation to other people and issues in their communities.

Ganz’s (26) framework, though frequently used as a tool for training and reflection in practice, draws on perspectives from across scholarly traditions, including the sociological study of institutionalism (27, 28) and from symbolic anthropology (16), linguistics (8), psychology (29), and critical education (30). Conceptualizations of narrative across these fields reveal common threads and point to other possible functions for narrative in grassroots community organizing. First, many view narrative as an element of group and collective identity that is constructed through interaction (16, 28, 31). The relationship between personal and collective identity, psychologists (32) suggest, is reciprocal and dialogic. Stories, they argue, “are created by people and in turn create us” (p. 216); shared narratives thus emerge as individuals interpret, modify, and re-tell community narratives to reflect their own experiences.

Second, public narratives can provide communal templates and meanings that enable groups of people to coordinate their behavior (33). Scholars of institutionalism consider, for instance, the cultural rules and cognitive structures that shape how individuals understand and enact their role within a larger system (34). Institutionalists see narrative “not as something that happens ‘inside’ a given box called organization, but as something that serves to construct the box itself” [(33), p. 276]. Organizations, in other words, exist only to the extent that the individuals constituting them enact a shared set of stories about what the organization does, how it does what it does, and the roles people play within it. As they are appropriated into our life stories, these community and organizational narratives shape peoples’ identities and behaviors (29).

Finally, the co-creation of narratives through dialogue can be a form of resistance to oppression (30). As instruments of domination, narratives provide “a sense of valuations, hierarchies, intersections, and privileges […] where we can place ourselves on a pecking order, raising the possibilities of narrative debasement and empowerment” [(35), p. 53]. Cultural myths and stories create symbolic boundaries between “us” and “them;” they identify who is valued as a speaker and who is not. Freire (30) asserted that, through dialogue, people reflect on themselves and the world, increasing the scope of their perception and depth of their awareness. They begin to see the world as a reality in progress, and understand that “the form of action they adopt is to a large extent a function of how they perceive themselves in the world” [(30), p. 83]. Thus, through dialogue, people can become partners in deconstructing the narratives that uphold oppression and in co-creating stories of a more just world.

How new narratives are constructed through, and help to galvanize, community action holds relevance for understandings of change processes that can alter structural determinants of health, yet few studies have examined the role of narrative construction in community action processes. Moreover, although Ganz’s model of public narrative has been broadly influential in scholarship [e.g. (36),] and in practice across a variety of domains (37), it has rarely been used to analyze narrative change in the specific contexts from which it was derived—grassroots community organizing initiatives. This study explores the personal narratives and public narrative work of participants in grassroots community organizing initiatives, using the stories of *self, us,* and *now* as an analytic framework. Our research questions were exploratory, considering (1) whether stories of *self, us,* and *now* were evident in discussions with grassroots leaders in two community organizing initiatives, (2) and how these elements of public narrative change efforts appeared to be interrelated, and (3) what sorts of challenges leaders were encountering in their efforts to resist or complicate dominant narratives and construct new public narratives.

## Method

### Study contexts

Participants were recruited from leaders in two federated community organizing chapters: the Amos Project in Cincinnati and Metropolitan Organizing Strategy Enabling Strength (MOSES) in Detroit. Both the Amos Project and MOSES are classified as congregation-based community organizing efforts.

#### The Amos project in Cincinnati

The Amos Project is part of the Ohio Organizing Collaborative and is affiliated with the organizing network Faith in Action. They employ a faith-based model of organizing and are best known for their work on early childhood education in Cincinnati. In 2003, Cincinnati first launched efforts for a private preschool program, with the support of corporate leadership, the Chamber of Commerce, and the local United Way. In 2010 this early childhood education initiative shifted to a publicly funded effort after realizing a system of privately-operated programs would not work. After several years of effort, the City Council refused to fund Cincinnati preschools, despite poor kindergarten readiness scores and the second highest childhood poverty rate in the U.S. In 2014, the Amos Project began mobilizing thousands across the city in support of funding preschool programs, and settled on a ballot initiative, as required, to get public funding. The Amos Project realized that funding this effort would require an income tax increase to guarantee the fiscal support necessary, but the business community refused to support a tax increase. The Amos Project decided to hold a public meeting with Cincinnati’s top business leaders, asking them specific questions about their commitments to early childhood education. Most of the business leaders ceded to the Amos Project’s position and supported the tax increase. The vote by Cincinnatians supported the tax increase for preschool education by a 62 to 38 percent margin (14).

#### Moses in Detroit

Metropolitan Organizing Strategy Enabling Strength (MOSES) is an affiliate of the Gamaliel organizing network. Founded in 1997, MOSES evolved from work by 14 Detroit congregations in 1987 to re-open a city swimming pool that had been condemned (38). That initial effort operated from a social service theory of change, but over time many from that original group came to understand that most neighborhood problems came from concentrated poverty and the economic, social, and political ramifications of this poverty. Several clergy members from the original group participated in trainings by the Gamaliel Foundation, and then MOSES formed as a Gamliel affiliate in 1997.

### Participants and procedures

For both sites, we interviewed key leaders (35 in-depth interviews across both cities) about their experiences with community organizing and with specific issue campaigns. Community organizing initiatives use the term “leaders” to refer to their volunteer members, language that reflects their unique orientation toward cultivating grassroots leadership through horizontal (rather than hierarchical) membership structures. To select the leaders that we invited to participate in interviews, we first drew on the meeting participation/attendance data shared by the leaders of the Amos Project and MOSES. The meetings/participation dataset we relied on was incomplete, so we selected participants who had participated more than one time and sent this list to staff organizers in both cities. We then engaged the staff organizers in a series of conversations about this list, having them add and eliminate potential participants based on several factors. For instance, we wanted to ensure that we were speaking to enough participants who had been deeply involved and could speak to many of the nuances of organizing based on their personal experiences. These individuals were expected to have the greatest insights into the phenomenon under study; namely, the process by which grassroots community organizing initiatives create and amplify new narratives (39). We also wanted to ensure that we captured the diversity of the broader base of participants across numerous factors: race, age, geography, socioeconomic status, religious background, primary issue involvement, etc.

Once we had agreed on a list of participants to invite, staff organizers in both cities e-mailed local leaders to let them know that a member of the research team would be contacting them to take part in an interview and that the interview data collection process was important for the local organizing initiative to gain insights into its effectiveness. They also communicated that participation was completely voluntary, that decisions to participate or not to participate in interviews would not affect their relationship with the organizing initiative in any way, and that no identifying information would be released from the interview data. Potential participants were then contacted by members of the university research team, given more details on the purposes of the interviews and the study procedures, and invited to schedule a time to complete the interview, typically over Zoom. A total of 64 participants were contacted in Cincinnati, of which 26 were ultimately interviewed. In Detroit, 14 participants were contacted, and nine were interviewed. Nonresponse could have been due to several factors, including personal challenges introduced by the pandemic (interviews and recruitment took place in the summer of 2020) or incorrect contact information. Additionally, some of the people we contacted responded with concerns that they were not knowledgeable enough about the organization to participate in an interview. Although most of those individuals ultimately did participate, it is likely that some nonresponse was due to similar concerns.

Interviews were semi-structured, meaning that the interviewer varied the wording and order of questions based on the flow of the conversation. Nevertheless, for all interviews, interviewers relied on a protocol with topics, prompts, and examples of question wording to help guide the conversation. Participants were asked about their general experience with community organizing, local issues that they had worked on, and narratives relating to those issues (e.g., “are there narratives in the community that influence/hinder your work on this issue?”; “are there examples of phrases, words, or images used to convey these narratives?”).

Table 1 summarizes participants’ demographic information. Questions about participants’ demographics were asked at the end of the interview, and due to the semi-structured nature of the interviews, these questions did not always get asked. Of the 35 interviews ultimately conducted, demographic information was therefore collected in 31 (89%). No participants refused to provide demographic information.

**Table 1:** Demographic characteristics of study participants.

Site	Cincinnati	Detroit	Total
Sampling			
Contacted	64	14	78
Interviewed	26	9	35
Demographics collected	22	9	31
Racial/ethnic identity			
Black	7 (32%)	5 (56%)	12 (39%)
White	12 (55%)	4 (44%)	16 (52%)
Jewish	1 (4%)	0 (0%)	1 (3%)
Asian American	1 (4%)	0 (0%)	1 (3%)
Biracial	1 (4%)	0 (0%)	1 (3%)
Gender identity			
Woman	13 (59%)	5 (56%)	18 (58%)
Man	9 (41%)	4 (44%)	13 (42%)
Socioeconomic status			
Upper/upper-middle	8 (36%)	4 (44%)	12 (39%)
Middle	11 (50%)	2 (22%)	13 (42%)
Lower/lower-middle	3 (14%)	3 (33%)	6 (19%)
Average age (years)	56	59	57

Questions about racial/ethnic identity, gender identity, and socioeconomic status were all open-ended (i.e., participants were prompted to self-define these categories, not given a list to choose from). Percentages were calculated using the total number of participants from whom demographic data were collected. Summed percentages may not equal 100 due to rounding.

### Analytic approach

Audio recordings of interviews were professionally transcribed in their entirety and then analyzed using MaxQDA software for qualitative data analysis. Interviews were coded inductively and deductively. Emergent codes were both descriptive and interpretive, with descriptive codes primarily capturing site-specific target issues (e.g., water equity in Detroit, education equity in Cincinnati) and interpretive codes capturing such themes as participants’ theories of change, new and prevailing public narratives, and the work/operations of staff organizers at each site. Deductive coding used a framework that identifies the following elements of public narratives: stories of *self*, stories of *us*, and stories of *now* (26). Inductive codes are nested within these deductive categories.

## Results

### Stories of self

“Stories of self” communicate the values that define who we are. Ganz (26) explains that “we construct stories around choice points—moments when we faced a challenge, made a choice, experienced an outcome, and learned a moral. We communicate values that motivate us by selecting among those choice points, and recounting what happened” (p. 283). These stories of self are inherently social and relational, forming the individual threads that weave together to form collective stories. They communicate aspects of one’s personal identity, which are “nested” within collective stories drawn from our faith, friends and family, media, and other features of social life.

The stories that leaders shared in their interviews provided us with insights into the ways that they understand the social systems that have shaped their experiences, how they interpret those experiences, and how those experiences have led to greater commitments to community action. In describing their experience with organizing, leaders and participants shared stories of self that communicated why they felt motivated to serve others and pursue certain forms of social change.

#### Stories of transformative encounters

The ways that people understand their position in society are developed through interaction with others (40). Participants’ descriptions of their “stories of self” revealed moments in which participants became aware of their own oppressive internalized logics and decided to act against them. For one MOSES participant, this moment came at an organizing training hosted by a national federation of local organizing initiatives (The Gamaliel National Network).

The first agitation the first night that stuck with me the whole time, [… it] was, “would you rather be liked or respected?” … somehow I realized I really wanted to be respected and I was tired of trying to please people.

She went on to explain,

I wanted to be respected, I wanted to shape myself differently, and I was tired of being intimidated by people in power, because […] there were a lot of people [like that at my job], especially White male CEOs. So I had different stories, encounters [at my job] that to this day I bring with me, because it was about being disrespected as a Black woman. And that just stuck with me. Just the need to be respected was a stronger driving force to decide to break through that fear and that intimidation that I was experiencing.

This participant’s story reveals how, through her encounter with the organizers at the training, she became more attuned to the internalized logics of patriarchal White supremacy that led her to subjugate her own needs. The seemingly innocuous fear of being disliked was, in fact, a mechanism of social power that prevented her from acting against an unjust system. Perspectives gained through participation with MOSES enabled her to name and begin resisting this form of power. This became an inflection point in her development as a leader.

For White participants, transformation often emerged from encounters that led them to reconceptualize their racial selves and their relationship to White supremacy. Close relationships with people of color brought participants’ attention to the racial power operating not just “out there,” but within themselves. An Amos Project participant shared that this reckoning had been particularly profound for her family:

We have […] deep friendships with people of color and now hearing those stories, the old narrative just doesn’t work anymore. It’s a lie. So, just trying to … I don’t know. It’s really important now. Having […] Black friends who share what they’re going through, it’s like a must-do type of thing.

These reflections suggest that deepening her relationships with people of color created a sense of accountability to action to which her White privilege may otherwise have been a barrier. These relationships personalized the effects White supremacy, inextricably binding her community’s survival to racial justice. By sharing her story with her White friends and family, she wove a new narrative in which White supremacy was not only harmful to Black people, but to their collective community. Her story of self therefore reflected her call to action, and its retelling cultivated new understandings of racial justice in her community.

#### Stories of the self as a political being

Related to stories of transformative encounters were stories of self that revealed shifts in participants’ perceived relationships to political systems. For some, this involved gaining a better understanding of the ways that policy decisions affected their lives, which motivated them to affect those decisions. To quote a MOSES participant:

I also learned [from organizing] how much our overall government really, really affects the way we live and the outcome, and in terms of our living, the quality of our life and our being. Whereas before I really never gave that as much of a thought and how pervasive it is in our lives. [This] has been an opportunity for me to really see the face of democracy, what it is and what it is not on all levels from the top to the bottom.

This participant was one of several who discussed the notion of democracy and individuals’ roles within it. Another described “wak [ing] up to […] participation in the political system,” through “democracy training,” explaining that,

If I don’t participate, I’m still perpetuating a system. So, it’s more like you do have a choice. You can vote no on this and say that you believe the current strategy gets that done. Or you can vote no and say, this is not important to get done. Or you’ve got to vote “yes” or find a way true enough to get to “yes.”

In this narrative, the individual is framed as a political being—as affected by political decisions, as having a voice in how those decisions are made, and as having a responsibility to use their voice to inform those decisions. Rather than a form of political neutrality, democratic nonparticipation is framed by this participant as a *choice* to perpetuate existing systems rather than disrupting them. This narrative holds individuals accountable to political decision-making whether or not they participate in voting and other democratic actions which, in turn, suggests that members of a political community are accountable to one another.

#### Storytelling as a relational practice

Sharing one’s personal story was seen as central to building the relationships that form the foundation of collective action. When asked about their personal story, a staff organizer explained that “it’s really to build relationship and rapport with people when I share my story.” This and other participants framed storytelling, not as a strategy used once to build a relationship, but as an ongoing practice that sustained long-term relationships:

I think my story evolves at different times, (A) because my story is always changing, but (B) because I think it's at the heart of my story to really build connection with people. And once I have that relationship with people, then whatever issue pops up, issues are going to come and go. I want to have a long-term developmental relationship with somebody so that we can work for however long[.] The issues are always going to change, so it doesn't really matter to me what the issues are. The heart of it is people connecting around, “we know we want to change things.”

This quote captures the dynamic nature of personal development, with repeated sharing of one’s story creating opportunities for individuals to express new understandings of themselves and their relationship to the world. At the same time, it highlights the role of storytelling practice in identifying and reaffirming shared values, enabling participants to remain committed to one another despite other changes.

Developing these types of deep and enduring relationships, participants suggested, required storytelling practices that were deep and authentic.

In this work, I’ve been able to tell my story. It’s something when people accept you for who you are […] there's a sense that people are listening. I can get caught up in your story and you can get caught up in their story. Even if you have other things on the agenda that you need to take care of, your story matters and I just find that to be important. Life-giving. It just seems like the right way to build an organization.

This same participant contrasted such “life-giving” storytelling practices with those at other organizations, much of which, he stated, “feels transactional […] it’s just extracting everything you can out of a person, out of a thing.” In this participant’s view, storytelling was a relational practice that cultivated new personal identities, sustained relationships, and provided the foundation for effective organization-building.

### Stories of us

While *stories of self* often emphasized the development of relationships or the role of personal interactions in shaping individuals’ experiences of the world, *stories of us* connected these relationships to a shared set of stories, collective experiences, and understandings of the world. As Ganz (26) explains, “all self stories are ‘nested,’ including fragments of other stories drawn from our culture, our faith, our parents, our friends, the movies we have seen, and the books we have read. Although individuals have their own stories, communities, movements, organizations, and nations weave collective stories out of distinct threads” (p. 285). These stories of us provide the foundation for collective action, with collective identities enabling groups to form and sustain their commitment to organizing processes.

#### Stories of political community

Like stories of self, stories of us articulated participants’ relationships to social and political systems. Stories of us were explicit, however, about the shared experiences that participants discovered through relationship-building, and about the power of organized groups to influence democratic systems. Reflecting on what they had learned from grassroots organizing, a participant explained,

I learned how important community and relationships are, and in building those relationships, how much our overall government really, really affects the way we live and the outcome in terms of our living, the quality of our life and our being. Whereas before I really never gave that as much of a thought and how pervasive it is in our lives.

This statement suggests that building relationships enabled individuals to recognize how their experience was linked to others’, and to begin viewing that collective experience as a *political* one.

A sense of shared political experience was then woven together with stories about the relationship between the community and elected leaders. To quote a MOSES participant,

It's just really amazing when you learn what the power of people can actually do. People feel so helpless of, “We’ve tried this before. We've talked to him. They come out, and they talk to us, and they shake our hands. One of them wants to be elected. And then when they get into office, you never see them again.” It’s like, “No, it's time to hold people accountable.” This is what we will tell them in these group meetings. “It’s time to hold people accountable. It’s time to make them earn their dollars. It’s time to make them represent us because that’s why they have the job.”

This story of *us* intentionally shifts narratives about helplessness to interconnected narratives of collective efficacy and mattering. In these new narratives, elected leaders have a responsibility to act in their constituents’ best interests, and citizens have the power to hold them accountable to that responsibility. Reframing political leaders as accountable to public good paved the way for feelings of indignance and group solidarity that overcame feelings of hopelessness and apathy. Such narratives also incorporated a call to act in democratic processes: “We have to be very vigilant, very involved and very engaged in what’s going on in our communities, what municipalities are doing, what local officials, authorities are doing, what our commissioners are doing.”

#### Stories of empowering settings

Stories of *us* communicated features of community organizing that participants considered to be central to the organization’s identity and to members’ experience. Central to several of these stories was an emphasis on building members’ skills and capacities. These processes were framed as inherently social, with organizing providing a setting in which participants could teach, learn, mentor, and support one another in becoming leaders and creating the change that they wanted to see in their communities. As one participant explained:

The reason that organizing is so important is because people come and go, you have newer people who know something is not right, but they don't have the language; they don’t have the access to the data; they don’t have the support to figure out how to address what they see as an issue in their communities. And groups like MOSES provide that support, provide that language, so that you can back up what you say, what you understand to be wrong. Now you can find a source to identify the data so that you know which you're saying is right, and it’s not just what you feel, but there are facts to back that up.

This participant’s observations show the important role that MOSES plays in supporting participants to develop language through which to understand issues in their communities and their relationship to those issues. People need narratives to make sense of their experiences in the world, and this participant suggests that organizing provides the space in which these new narratives can be constructed. These shared understandings of how the world works provide a foundation through which members become active and critical participants in social and political processes.

Like others, this participant also underlined the interconnectedness of what Ganz (26) terms the *head,* the *heart,* and the *hands:* the analysis, the emotion, and the skills that drive and enable action. One participant explained that “Oppression, depression, racism, it’s a building block for all of it, it is. It’s like, keep the people ignorant and you can control them, give them some education and they’ll just lash out and ask for the world.” The processes of building new skills, developing new political subjectivities, and constructing shared meanings were thus viewed as inextricably connected and central to the process of building relationships and powerful organizations.

#### Stories of collective action

Stories of us also communicated the norms and social regularities that guided the groups’ collective action, linking new political subjectivities to organized strategic actions that could be taken to influence structures and systems. As one participant explained,

Going to events, or we call it “coffee hour,” or when a legislator or an important person opens up to the community to go to, that should be encouraged to go to, and not just one person. [For example,] [t] he municipality of Pittsfield Township is having an open house about public safety, and so […] we want at least three people to go and to hear them out. So encouraging doing those things, bringing that information back and talking about it.

Organizing, in other words, created organizational norms and expectations around democratic participation that guided members in their collective actions. These norms and expectations formed the basis of their power, enabling the group to demonstrate vigilance and commitment to change.

### Stories of now

Stories of *now* combine strategy and story in a call to action (26). They articulate the challenges we face now, communicating how “we are called on to act because of our legacy and who we have become, and the action that we take now can shape our desired future” [(26), p. 286]. Participants shared stories of now that related both to specific initiatives and actions, such as water shutoffs, and to pivotal moments on their own pathways to leadership.

#### Urgency

Urgency, Ganz (26) explains, can overcome inertia, or the tendency to operate by habit and respond in “programmed” or automatic ways to events in our day-to-day lives. Urgency is about making something a high priority, and initiatives in both Cincinnati and Detroit developed narratives that emphasized the urgency of certain issues. Participants in Detroit explained, for instance, how underscoring the importance of water for sanitation during the COVID-19 pandemic enabled them to negotiate a moratorium on water shutoffs until 2023.

Say, that person has to go out during the pandemic to get bottled water. That’s an opportunity for disease to be spread. So that really gave us a whole other way to frame it, and we use that. Like, water is a necessity, when we need to stay home and to protect ourselves and others.

This was a significant achievement in their longer water equity campaign. Likewise, an initiative in Cincinnati was able to elevate the urgency of certain goals by framing them in terms of the COVID-19 pandemic. The Heights Movement, with support from the Amos Project, has recently reinvigorated a decades-long effort to remove a police gun range from the neighborhood. To quote one participant,

Nobody can argue against the merits of the case and why this should be done. All it requires is for you to hear once, and you’re like “this is not right.” It's terrible. And, six days a week, it's safe to say there’s no less … on a good day, you wouldn't hear anything less than let’s call it 400 gunshots. That’s a good day. So, if you take that, four times six, that’s 2400 gunshots a week times 52 weeks, it's over 100,000 gunshots a year. You extrapolate that out for … And of course, it got worse through the militarization of the police so we're talking about crack era on, it's been horrific.

The Heights Movement and the Amos Project have worked to amplify the story, emphasizing the harmful effects on children who were attending school remotely during the pandemic.

You can’t argue without just looking like a complete and utter ass. Excuse the language. You can't argue against harming kids, and then the facts that it's COVID and kids were working from homes, but you're still firing guns in the middle of the day, so kids are literally on Zoom and you hear the gunshots in the back and they're trying to focus on class.

Although a decision has not yet been made about relocating the gun range, framing the issue as related to COVID-19 has created the possibility of utilizing Hamilton County pandemic relief funds. This is yet another example of organizing responding to ever-changing landscapes of political opportunity.

#### The world as it should be

Regardless of the specific issue at hand, stories of now often centered the notion that the world could be changed, and that the community could change it. A staff organizer explained, for instance, that

The heart of [organizing] is people connecting around, “we know we want to change things.” Issues are always going to change, but it's always going to evolve around this idea of the structural racism that we face in Detroit, the inequity that is just really baked into our day-to-day lives that we don’t even see. It's just so obvious. It’s so, it’s so clear, but so, but people don’t see it as, as anything but kind of normal.

**Figure 1:**
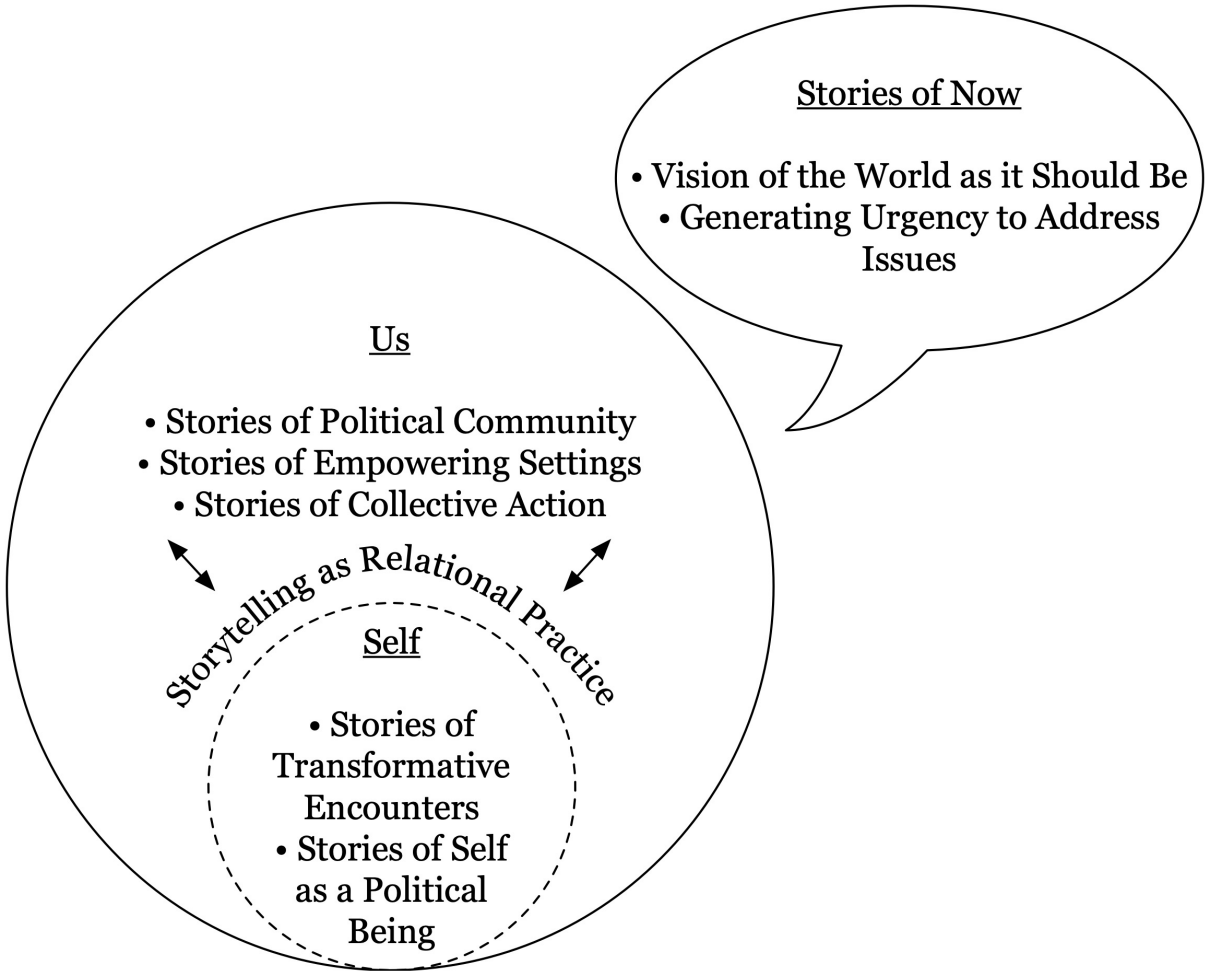
Illustration of the major themes identified in this study.

New narratives illuminated the injustices in day-to-day life, challenging the normalcy of such phenomena as poor education, water shutoffs, and incarceration. Doing so re-framed the ways that residents interpreted and responded to issues, and created space for the development of new relationships to democracy. As one participant explained, “they are training us that whatever comes at us and our community, we can stand up and say, ‘No, this is wrong.’ Or ‘There’s another way to do it.’ We can have a voice, we matter.” Figure 1 summarizes the conceptual relationship of these stories of now to stories of self and stories of us.

### Challenges

The description of work by the Amos Project and MOSES thus far has emphasized elements of organizing that have largely been effective at building and exercising power. We have presented descriptive evidence on how narratives are constructed and modified through the relational processes of community organizing, highlighting the role of narrative in building, sustaining, and guiding grassroots organizations. This study also provided insights into several challenges and tensions that can hinder organizing’s effectiveness and/or it impacts, and several other factors that can limit the potential of organizing to work with other groups in pursuit of improvements in public health. We therefore sought to identify some of the challenges that these organizing initiatives faced during the study with the hope that insights gleaned from the data in this project may be instructive for similar efforts in the future.

It is worth noting that both Detroit and Cincinnati are highly racially segregated cities, and organizing in both cities must contend with this reality. Some White participants described work related to raising awareness around racial justice. Yet, it was clear that some of these participants were not developing into leaders in the organizing initiative. Several White participants, for instance, did not sustain their involvement in community organizing after specific initiatives, suggesting that their interest was issue-specific rather than rooted in a deep and sustained commitment to building power. Others attributed their reduced participation to the departure of a specific leader, pointing to an unsustainable leader-centric structure rather than one of dispersed leadership. Certain participants cited these and other trends in critiques of efforts to organize White churches, voicing a sense that Black organizers were tired of their work being co-opted by these groups. These perspectives speak to the challenge that segregated cities face in building multiracial coalitions.

We also spoke with several White faith leaders who, despite expressing support for the organizing initiative and describing relationships with some leaders, positioned their own work as adjacent to that of the organizing initiative. Many expressed discomfort with the idea of conflict and organized political action, indicating that confrontation was not the way to change hearts and minds. One, for example, explained:

So that’s what I think that within organizing, we're trying to build those connections. And those connections are being built. […] we’re then moving into a place of what gifts we bring to the table. I mean that’s what [community development] is. It’s how do you prepare the table? What gifts you bring to the table, and then out of those, not looking at the needs, but how do we apply the assets, and so in community organizing, if there’s an issue, if we’ve got relationships then we can kind of then see the connective tissue, and then how do we work that connective tissue. And that’s part of the reason I haven’t been that involved with [the organizing initiative] on the inside because I think that there’s a power in the relationship.

This faith leader elevates a theory of change that emphasizes cooperation and collaboration, both within the organization and with decision-makers in the community. Other leaders expressed similar views, suggesting, for instance, that leaders could be persuaded using appeals to shared values and faith. This contrasts models for organizing that begin from the assumption that any effort to transform the status quo will be met with resistance. Conflict, in other words, is inevitable in the pursuit of equity-focused change.

These perspectives from participants reveal tensions in efforts to organize White faith communities in particular. However, these challenges are important to highlight in the context of the current project, as they illustrate the entrenched worldview among some faith leaders that conflictual approaches to achieving change will not work and that cooperation is what produces effective change. This worldview is another form of narrative that can override the perspective and historical evidence that organizers may provide (e.g., the civil rights movement) about how community change happens. Finally, the adherence to a worldview that favors cooperation over conflict exemplified in these quotes may reflect an impact of COVID in that in times of great uncertainty, people tend to cling to the status quo (41, 42).

## Discussion

Changing narratives is an important strategy for promoting public health and health equity. Narrative change efforts are generally viewed as improving upon previous models of health communication by “shap [ing] hearts and minds” [(10), p. 8] and changing behavior by providing “an alternative worldview” [(5), p. 497]. At the same time, scholars across disciplines characterize narrative as playing an important role in building and sustaining organizations (33), perpetuating or disrupting dominant ideologies (43), cultivating community identity (44), and linking cognition to action (26).

This study qualitatively examined the narratives and processes of narrative construction among grassroots organizing leaders in two U.S. cities: Cincinnati, OH and Detroit, MI. Analysis used Ganz’s (26) framework for *public narrative* as consisting of stories of *self*, stories of *us*, and stories of *now*, considering whether these stories were evident and, if so, how they appeared to be interrelated. We additionally explored the types of challenges leaders encountered in their efforts to resist or complicate dominant narratives and construct new public narratives. The results of this study suggest that the construction and sharing of public narratives plays a role in community power-building by facilitating psychological changes experienced by members in organizing, cultivating the relationships that sustain organizations, and communicating the expectations and norms that guide organizational actions. They indicate, furthermore, that the process of narrative change is deeply relational and dynamic, a finding that challenges assumptions of some programmatic or top-down approaches to narrative intervention.

First we examined leaders’ descriptions of stories of *self,* which communicate the values that define who we are (26). Several participants’ personal stories centered moments of personal transformation, highlighting in particular the social settings and personal relationships that facilitated shifts in their personal identities. For several participants, recognizing one’s racial self was a key component in this process. A White participant, for instance, experienced what Gupta (17) describes as “a sense of other-in-self,” which “moves people in positions of relative privilege to comprehend their role as fighting for their own freedom, expressing a self who is accountable to themselves as well as others” (p. 14). Other participants described becoming aware of internalized White supremacist and other logics of domination, suggesting that, by naming those influences, they could intentionally pursue new ways of thinking and acting. In several cases, transformative encounters built more agentic subjectivities, as participants felt an increased sense of responsibility or capacity to change their circumstances. This was particularly true when transformative encounters were tied to new understandings of one’s relationship to democracy and political processes. Participants’ personal stories suggested that viewing oneself as a political being is often linked to an increased sense of political agency, viewing full participation in democracy as necessitating action. Such narratives countered the dominant narratives of powerlessness and political apathy that prevented residents from acting to change inequitable systems and structures (13, 17).

Stories of self were also understood to be deeply relational and dynamic, with storytelling viewed as a practice that can aid in self-reflection while building and sustaining relationships and organizations. Personal stories, when told and re-told over time, incorporate one’s new understandings of the world and one’s place within it (32) and communicate those understandings with others. Acknowledging changes to one’s personal story can play a part in growth and development, and the sharing of these changing stories can help to build and sustain interpersonal relationships. These findings accord with previous studies highlighting the importance of personal storytelling in building relationships of trust and accountability that form the foundation of powerful grassroots organizations (23, 24). They further underscore the potential psychological benefits of personal storytelling, which one participant described as “life-giving.” Personal storytelling can play key roles in generating power through relationship development, through personal healing (45), and through agentic identity development.

Second, stories of *us* communicated the shared identities and collective practices that were developed and strengthened through participation in organizing activities. Participants shared stories of *what* the group was capable of and *how* they achieved their goals, communicating both a sense of collective ability to influence social and political systems and a set of cultural values and practices that guided their actions. Constructed and shaped through the sharing of personal stories, collective narratives reflected participants’ personal experiences and values as well as their shared experiences, understandings of the group and its values, and the community’s place in the world (16). These new narratives were often *counter* to internalized cultural narratives about the community which, for instance, framed community members as powerless to change community conditions and ignored by political decision-makers (29). New narratives, developed as members participated in organizational processes of research and action and were exposed to organizational narratives centering collective efficacy, democratic accountability, and political participation, reflected hope for the future (26). New narratives therefore expanded members’ perceptions of what futures were possible for themselves and their communities, providing the foundation for action (17, 46).

Narratives about the organization and community also provided a template for how one’s individual capacities and agency could be translated into powerful collective actions. This could be seen, for instance, in the expectation that multiple members of the organization attend any public meeting or appearance by a decision-maker; by communicating organizational norms, such narratives guided members in coordinated actions that demonstrated power to other organizations and decision-makers in the community. Sustained and consistent member participation in organizational activities is the lifeblood of grassroots community organizing initiatives, solidifying “the development of interorganizational relationships, largely through the promise of reward for organizational entities who cooperate and punishment for those who do not” [(15), p. 738]. As individual members internalized and acted upon organizational narratives such as “remaining vigilant” and holding political leaders accountable, their actions manifested in coherent organizational actions that demonstrated power to other entities.

Third, stories of *now* particularly highlighted the strategies undertaken at each site to build energy around a specific issue. Both the Amos Project and MOSES were able to generate a sense of *urgency* around specific issues, re-framing much larger challenges such as water and education equity in terms of the immediate threat of COVID-19. Narratives were constructed and disseminated that articulated the crisis and presented decision-makers with a choice, encouraging them to find the courage, hope, and empathy to respond (26). For the Amos Project, this story centered around children’s education and welfare; for MOSES, this story was about public health and water as a human right. Both offered a credible vision for how to proceed, with MOSES calling for a memorandum on water shutoffs and the Amos Project calling for relocation of the gun range. In each case, a clear decision was presented between right and wrong, between action and inaction. These elements, Ganz (26) suggests, create a story in which a decision-maker (often the carefully selected target of action by community organizing initiatives) is the protagonist, faced, perhaps, with a decision between what is right and what is easy. The story of now gives the decision-maker an opportunity to be the hero of the story, and a clear path for how to do so.

In addition to nimbly reframing issues in response to crises such as COVID-19, stories of *now* also reframed taken-for-granted inequitable conditions as unjust and intolerable. Ganz (26) notes that the major “action inhibitor” is “inertia—operating by habit and not paying attention. We process most of the information that comes our way on ‘autopilot,’ and we respond as programmed” (p. 277). Programmed responses often reflect the interests of the powerful, manifestations of internalized oppressive ideologies (43, 47). In stories of *now,* inertia is disrupted through re-framing of “normal” phenomena as unjust and immoral. New stories precipitate shifts from apathy, fear, and self-doubt to anger, hope, and confidence in one’s perception of reality (26). For both MOSES and the Amos Project, much of this work involved making the effects of White supremacy visible and applying racial justice frames to issues that mattered to the community.

Finally, our results also highlight the challenges of building multiracial coalitions, particularly in highly segregated contexts. Many White participants’ personal stories highlighted moments of racial awakening, which in some cases led them to participate as leaders in a racial reconciliation training or engage differently with their friends and family. However, it was often the case that these participants’ increased awareness of White privilege and racial injustice was not connected to feelings of *other-in-self* (17), but to a feeling of being separate from—yet morally obligated to—the other. While feelings of *other-in-self* in some cases led to a sense that racial justice was central to collective human survival, in other cases separation and obligation enabled White participants to pursue racial justice as a form of self-actualization rather than as an urgent imperative for systemic changes.

Despite these challenges, both of the organizing initiatives that took part in this study have strong track records of building and exercising power. Whether limiting water shut-offs, organizing to stop gunfire from a range adjacent to an elementary school, increasing the minimum wage, altering policies about detaining suspected immigration violations, or advancing preschool education, these organizing initiatives have demonstrated a set of skills and strategies for changing policies that advance public health goals. These skills and strategies are not fixed practices that can be packaged and disseminated to other communities and successfully applied if executed with fidelity. In contrast, what emerged from these data was that successful change is a labor-intensive process and one that requires the development of leaders (stories of self) and the cultivation of collective structures (stories of us) capable of acting with power to effect change with urgency (stories of now).

Findings from this study should be interpreted considering several limitations. Some of the observed dynamics may be particular to urban areas in the American Midwest, for instance, or to the unique period when this study was conducted (during lockdowns due to the COVID-19 pandemic and racial justice uprisings following the murder of George Floyd). Furthermore, interviews were only conducted with a non-representative subset of the leaders in each organizing initiative. The interviews conducted for this study nevertheless capture the nuance and sophistication in methods organizers use to cultivate understandings and articulate community values that become the anchor for narrative change efforts. They illustrate the complexity and multi-layered quality of narrative change work that is conducted within grassroots organizing initiatives.

Finally, this study’s findings suggest several avenues for ongoing research and for enhancing efforts to link narrative change efforts to meaningful progress on addressing the social determinants of health. First, this study suggests the value of developing multi-faceted narratives as part of community-driven change efforts. In programmatic narrative change interventions, there is often an emphasis on identifying optimal terminology or tailoring particular messages to targeted audiences. In contrast, the narrative change processes examined in this study engaged people in building cohesive collective structures and acting to exercise power—steps that are rarely addressed in studies of narrative change work. Scholars should continue to investigate narrative change processes in grassroots community organizing initiatives and seek greater understanding of how some groups are meeting the challenges inherent in weaving diverse participants’ personal stories together into stories of collectivities united in their pursuit of changes that can promote health equity.

## Data availability statement

The raw data supporting the conclusions of this article will be made available by the authors, without undue reservation.

## Ethics statement

The studies involving human participants were reviewed and approved by Vanderbilt University Institutional Review Board. The patients/participants provided their written informed consent to participate in this study.

## Author contributions

PS and BC designed and planned the study and supervised the work. KH and HF conducted the interviews. KH led the analysis with support from GC-W. KH, BC, HF, PS, and GC-W discussed the analysis and results. KH drafted the manuscript. KH and BC performed major revisions. All authors read and approved the final manuscript.

## Funding

Funding for this research was provided to the National Network of Public Health Institutes (NNPHI) through a Cooperative Agreement with the Centers for Disease Control and Prevention of the U.S. Department of Health and Human Services (HHS) as part of a financial assistance award (CDC – 6 NU1ROT000016–01-02) totaling $2,900,000.

## Conflict of interest

The authors declare that the research was conducted in the absence of any commercial or financial relationships that could be construed as a potential conflict of interest.

## Publisher’s note

All claims expressed in this article are solely those of the authors and do not necessarily represent those of their affiliated organizations, or those of the publisher, the editors and the reviewers. Any product that may be evaluated in this article, or claim that may be made by its manufacturer, is not guaranteed or endorsed by the publisher.
